# A Rare Case of Bleeding Epiphrenic Esophageal Diverticulum From Arteriovenous Malformations

**DOI:** 10.1177/2324709620901942

**Published:** 2020-01-23

**Authors:** Franklin L. Thelmo, Harvey Guttmann, Waqas Ullah, Ahmad Arslan, Eugen Brailoiu

**Affiliations:** 1 Abington Memorial Hospital—Jefferson Health, Abington, PA, USA; 2 Temple University Hospital, Philadelphia, PA, USA

**Keywords:** epiphrenic diverticulum, gastrointestinal, esophageal disease, endoscopy, argon plasma coagulation, esophageal diverticulum

## Abstract

Epiphrenic esophageal diverticula (EED) is a rare condition that usually presents with dysphagia in patients with a known motility disorder. In this article, we present a unique case of EED presenting with hemoptysis with clinical workup negative for any pulmonary pathology. Esophagogastroduodenoscopy revealed arteriovenous malformations within the EED successfully managed with argon plasma coagulation (APC), leading to a resolution of the patient’s symptoms.

## Introduction

Epiphrenic esophageal diverticulum (EED) is an outpouching of some or all layers of the esophagus and is quite rare, accounting for only 0.0015% to 2% of cases.^1,2^ It occurs with a slight male predominance, and the average age of diagnosis is between 60 and 70 years.^3^ Diverticula <5 cm is more likely to remain asymptomatic.^4^ When symptomatic, they tend to present with dysphagia, regurgitation, and epigastric pain.^1-3,5^ However, there have been no reports in the literature in which a patient had a bleeding EED due to arteriovenous malformations (AVMs).^5,6^ Both EED and esophageal AVMs are rare phenomena independently, and there is no description on a literature review of them occurring simultaneously. When an EED does occur, it tends to lie within 10 cm of the gastroesophageal junction and is generally right-sided.^1-3,5^

## Case Report

A 53-year-old male with a past medical history of myocardial infarction and gastroesophageal reflux disease (GERD) presented to the emergency department (ED) for regurgitation of blood and epigastric pain. The surgical team evaluated the patient, as the patient endorsed several episodes of regurgitation with approximately 200 mL of blood loss. The patient endorsed violent coughing during these episodes, and the working differential at the time was hemoptysis versus hematemesis. The patient had been taking 81 mg of aspirin and 40 mg of omeprazole for many years without any complications. He denied dysphagia, epigastric pain, headaches, shortness of breath, dyspnea on exertion, back pain, nausea, vomiting, diarrhea, and constipation. The patient had no history of gastric motility disorders or peptic ulcer disease. His GERD symptoms were mostly under control with the occasional flare of dyspepsia and scant regurgitation but not above his baseline. He had no changes to medications. He had no new diet, activities, travel, recent injuries, or sick contacts. He had a 20 pack per year smoking history and he quit 5 years ago, drank alcohol only on special occasions, and denied any history of drug use.

On physical examination, he was found to be uncomfortable but not in acute distress. Vital signs showed blood pressure was 143/91 mm Hg, pulse of 90 bpm, and afebrile at 37.1°C. His lungs were clear to auscultation bilaterally without rales or rhonchi; he had no increased work of breathing; his abdomen was soft, nondistended, and mildly tender in the epigastrium.

His laboratory tests showed a hemoglobin of 16.2 mg/dL in the ED, which dropped to 12.7 mg/dL afterward. The patient had a normal comprehensive metabolic panel, troponin, and creatine kinase. The patient had negative sputum culture and normal d-dimer. Computed tomography scan of chest showed mild right-sided pulmonary infiltrates.

**Figure 1.**
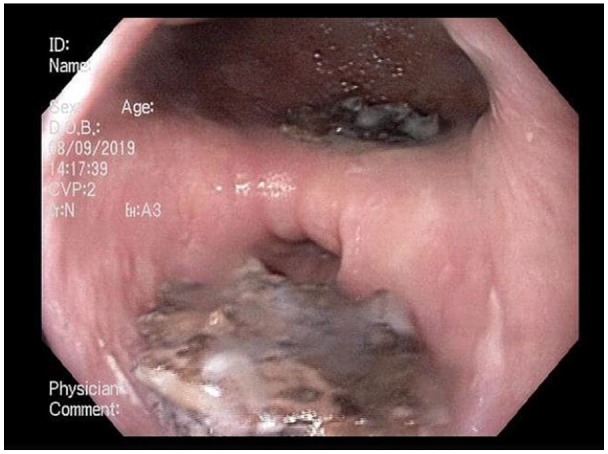
Epiphrenic esophageal diverticula with clot burden superior to the lower third of the esophageal lumen with clot burden.

**Figure 2.**
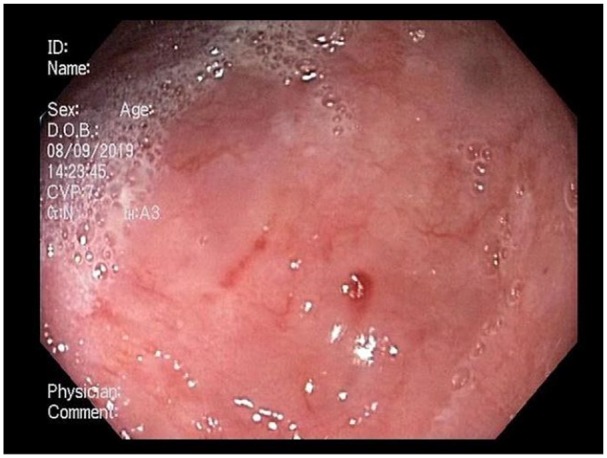
Adjacent arteriovenous malformations within the epiphrenic esophageal diverticula base.

**Figure 3.**
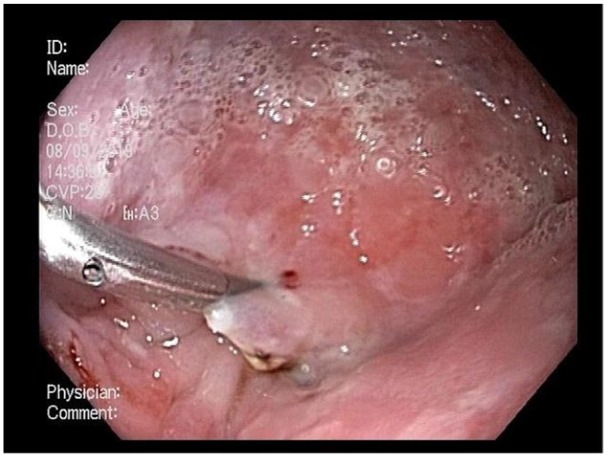
Application of argon plasma coagulation and endovascular clip atop the 2 arteriovenous malformations.

Continued hematemesis in the ED prompted a consultation for gastrointestinal (GI) specialists. GI had immediate concerns for peptic ulceration, esophageal varices, and Mallory-Weiss tears. The patient was prepared for esophagogastroduodenoscopy (EGD) with possible intervention. On EGD, the patient was found to have no signs of ulceration or complex tortuous esophageal anatomy. Further exploration revealed a large right-sided 10 × 10 cm nonbleeding EED located 4 cm from the gastroesophageal junction. A significant clot burden was noted in and around the EED (Figure 1). He also had 2 adjacent AVMs within the EED (Figure 2). The AVMs were treated with argon plasma coagulation (VIO 300D, Erbe USA, Inc, Marietta, GA) on a low-energy setting, and an endovascular clip was placed (Figure 3). Biopsies were taken from the middle third and lower third of the esophagus. Biopsies revealed benign squamous and glandular mucosa with moderate acute and chronic inflammation and were negative for intestinal metaplasia and dysplasia. The procedure was completed without any complication, and the patient awoke from sedation without incident.

Following the procedure, the patient was discharged to home with stable hemoglobin and no further signs of symptoms or bleeding on follow-up. He was recommended to follow-up with the outpatient GI office within 2 weeks of discharge and recommended to have annual EGDs to assess for the progression of the EED. He was referred to surgical colleagues for further evaluation. He was informed that surgical intervention is unnecessary if he had no further episodes of hematemesis and remained asymptomatic.

## Differential Diagnosis

During the patient’s emergency room visit, the differential diagnosis included peptic ulcer disease, esophageal varices, gastritis, gastroenteritis, pneumonia, pulmonary embolism, and posterior epistaxis.

## Treatment

EGD was used to remove the clot burden within the EED and lower esophagus. APC was applied to the 2 AVMs, and an endovascular clip was placed over them. Biopsies were taken of the local esophageal tissue and gastric antrum. The patient was maintained on his normal proton pump inhibitor on discharge and advised to follow-up with the outpatient GI office in the next few weeks.

## Outcome and Follow-up

On outpatient GI follow-up, 1 month after the patient endorsed no further issues of severe epigastric pain or hematemesis. He was recommended to have an EGD in 1 year, and he is being evaluated by surgery for diverticulectomy at this time.

## Discussion

EED is a rare disease in its own right with an annual occurrence of roughly 1 in 500 000.^1-3,6^ The development of AVMs within the esophagus is even less commonly encountered with the overall prevalence of upper GI bleed due to AVMs being 2% to 5% and the esophagus being the source of AVMs as low as 0.5%.^5^ Our case represents a rare combination of a patient with EED and significant hematemesis due to the development of AVMs within the EED. Up to 80% of patients with EED will be asymptomatic.^6^ Patients with symptomatic EED initially present with dysphagia, nonbloody regurgitation, and epigastric pain. This is the second-ever case of a bleeding EED due to an AVM and the first described in the literature of hemostasis being achieved using APC and endovascular clipping for it.^5,7^

We performed a comprehensive literature search of all case reports of bleeding esophageal epiphrenic diverticulum through PubMed, without a time filter or language barrier. The search terms included “bleeding epiphrenic diverticulum,” “epiphrenic esophageal diverticulum,” and “epiphrenic diverticulum and AVMs.” We found 8 case reports recording EED that presented initially with large amounts of hematemesis. Case reports were reviewed, and characteristic summaries can be found in Table 1.

**Table 1.** Characteristics of Bleeding EED Cases Reported.

Authors	Age/Sex	Presentation	Finding on Imaging	Cause of Bleed	Outcome
Tse and Parikh^7^	83/male	Hematemesis, melena, lightheadedness	Epiphrenic diverticulum	Oozing of local vessel	Hemostatic clips and discharged
Ballehaninna et al^8^	61/male	Hematemesis and melena	Epiphrenic diverticulum 22 cm from incisors	Ulceration and fistula	Gastrostomy tube led to resolution in 3 months
Garcia et al^9^	86/female	Hematemesis	Epiphrenic diverticulum 2 cm from Z-line	Unidentified	PPI and conservative management, discharged home
Chen et al^10^	47/male	Unconscious following hematemesis	Epiphrenic diverticulum	Ulceration into the left gastric artery in setting of coagulopathy	Death
Hoxie et al^11^	51/male	Hemoptysis	Epiphrenic diverticulum	Ulceration	Bleeding ceased, patient refused further intervention
Abul-Khair et al^12^	49/female	Dysphagia and hematemesis	Epiphrenic diverticulum	Ulcerating crypt	Diverticulotomy and myotomy led to resolution
Bozorgi et al^13^	27/female	Weakness, hematemesis, melena	Epiphrenic diverticulum with occupying lesion	Large esophageal leiomyoma	Surgical resection and discharge
Sivanes and Chang^14^	87/male	Syncope and melena	Epiphrenic diverticulum	Unable to identify	Self-resolved, advised to stop aspirin
Turan et al^15^	63/male	Hematemesis	Epiphrenic diverticulum 28 cm from incisors	Dieulafoy’s lesion	Resolution following Sengstaken-Blakemore tube insertion

Abbreviations: EED, epiphrenic esophageal diverticula; PPI, proton pump inhibitor.

Table 1 displays 8 different case presentations from authors via MeSH in which patients with epiphrenic diverticula first presented with symptoms due to bleeding. Of the 8 cases, 5 were found in males and 3 in females, consistent with the male predominance of EED.^1-3,12,13^ Of the 8 cases, an identifiable reason could not be found as a cause for the bleeding for only 2 cases.^9,14^ In 4 of the 8 cases, the bleeding EED was due to ulceration within the EED.^8,10-12^ In only 1 case was a patient found to have such profound bleeding that it led to their death; all other patients were discharged home stably.^10^ In only 1 case was a direct vascular lesion noted.^15^ In only 1 case was the outcome of the patient’s disease unknown as they refused to follow-up further with investigators.^11^

EED is usually diagnosed in patients following a prolonged history of dysphagia, epigastric pain, and regurgitation or in the setting of a known history of gastric motility disorders. The diagnosis is made via a Barium swallow study and EGD. EED has also been diagnosed with EGD in asymptomatic patients who are having the procedure for other reasons.^6^

On discovery of the diverticula, patients should be evaluated for further underlying motility disorder and have an endoscopy to evaluate the integrity of the lesion regularly. Though rare, squamous cell carcinoma has been recorded within diverticula and can be a cause of bleeds similar to our patient; thus, lesion biopsy is paramount.^1,2,6^ There is a multitude of laparoscopic surgical techniques available for resection of an epiphrenic diverticulum, but they are not without significant morbidity and mortality. Patients may opt for more conservative management with annual or biennial follow-up with endoscopy as issues arise.^6^ Patients without concerning pathology who do not have any symptoms do not need further workup. Our patient had clot burden removal, regional tissue biopsy, APC application to the 2 AVMs followed by endovascular clipping given the uncommon presentation of clinical instability.

Without any concerns for severe symptoms or malignancy, the prognosis is quite favorable with many patients requiring no further workup as most do not result in clinically significant disease.^6^ Regular proton pump inhibitor use, diet adjustment, and minimal nonsteroidal anti-inflammatory drug use can provide the basis for conservative management. For persistent symptoms requiring surgical intervention, laparoscopic or video-assisted thoracoscopic surgery are preferred to open procedures and will encompass a variety of fundoplication techniques with or without a myotomy based on the patient’s presence of GERD. Surgical intervention though remains a second-line option given the wide risk of mortality from the procedure.^2,3^
